# Integrated optofluidic-microfluidic twin channels: toward diverse application of lab-on-a-chip systems

**DOI:** 10.1038/srep19801

**Published:** 2016-01-29

**Authors:** Chao Lv, Hong Xia, Wei Guan, Yun-Lu Sun, Zhen-Nan Tian, Tong Jiang, Ying-Shuai Wang, Yong-Lai Zhang, Qi-Dai Chen, Katsuhiko Ariga, Yu-De Yu, Hong-Bo Sun

**Affiliations:** 1 State Key Laboratory on Integrated Optoelectronics, College of Electronic Science and Engineering, Jilin University, 2699 Qianjin Street, Changchun, 130012, People’s Republic of China; 2 College of Physics, Jilin University, 119 Jiefang Road, Changchun, 130023, People’s Republic of China; 3 International Center for Materials Nanoarchitectonics (MANA), National Institute for Materials Science (NIMS), 1-1 Namiki, Tsukuba, 305-0044 Japan; 4 Precursory Research for Embryonic Science and Technology (PRESTO) and Core Research for Evolutional Science and Technology (CREST), Japan Science and Technology Agency (JST), 4-1-8 Honcho, Kawaguchi, Japan; 5 State Key Laboratory on Integrated Optoelectronics, Institute of Semiconductors, Chinese Academy of Sciences, Beijing 100083, China

## Abstract

Optofluidics, which integrates microfluidics and micro-optical components, is crucial for optical sensing, fluorescence analysis, and cell detection. However, the realization of an integrated system from optofluidic manipulation and a microfluidic channel is often hampered by the lack of a universal substrate for achieving monolithic integration. In this study, we report on an integrated optofluidic-microfluidic twin channels chip fabricated by one-time exposure photolithography, in which the twin microchannels on both surfaces of the substrate were exactly aligned in the vertical direction. The twin microchannels can be controlled independently, meaning that fluids could flow through both microchannels simultaneously without interfering with each other. As representative examples, a tunable hydrogel microlens was integrated into the optofluidic channel by femtosecond laser direct writing, which responds to the salt solution concentration and could be used to detect the microstructure at different depths. The integration of such optofluidic and microfluidic channels provides an opportunity to apply optofluidic detection practically and may lead to great promise for the integration and miniaturization of Lab-on-a-Chip systems.

Using a lab-on-a-chip with microfluidic channels and active microfluidic devices, conventional processes, such as chemical or biological sample preparation and optical or electronic analysis, normally performed in a lab can be miniaturized and performed on a single chip^1^,^2^,^3^. Due to the significant benefits of the scaling down of size, minimal consumption of reagents, and reduced manufacturing costs, lab-on-a-chip has potential applications in chemical, biological and medical analyses^4^,^5^,^6^. To realize different optical components and to create a highly versatile microsystem, optofluidics, which integrates microfluidics and micro-optical components that have previously been used outside the chip can now be integrated into a micro- or nanoscale chip, provides a unique solution for generating, manipulating, and controlling optical signals on a chip-based platform^7^,^8^,^9^,^10^. Until now, the majority of optofluidic research has been focused on the fabrication of optical components controllable by fluidics, including optical lenses, waveguides and a light source^11^,^12^,^13^,^14^,^15^. The critical goal of creating optical components is to build an integrated system of devices in which the optofluidic component is used to detect, analyze, and physically manipulate micro-objects or nano-objects in the adjacent fluidic environment^16^,^17^,^18^,^19^,^20^. However, for the above-mentioned goals, only particle trapping studies by optofluidic manipulation have been reported. Schmidt *et al*. showed an optofluidic chip as an all-optical particle concentrator based on a fully planar geometry utilizing counterpropagating liquid-core waveguide modes to form a loss-based optical trap^21^. Meanwhile, to manipulate nanoscopic matter precisely, Yang *et al*. reported on the optofluidic trapping and transport of 75-nm dielectric nanoparticles and λ-DNA molecules using subwavelength liquid-core slot waveguides^22^.

In the present microfluidic channel system, the dominant technology of optical micro-detection and analysis is the introduction of fabricated micro-optical components rather than optofluidic devices, which adjust the optical properties of optical elements by controlling fluids^23^,^24^. Various 2D and 3D micro-optical components can be flexibly integrated into a microfluidic channel by femtosecond laser direct writing to improve the portability of lab-on-a-chip systems^25^,^26^. Stimuli-responsive hydrogels are a class of cross-linked polymers that have the ability to absorb water, which can undergo phase transitions, wherein an external stimulus, such as pH, temperature, ion concentration, or light, gives rise to distinct volumetric changes in these hydrogels^27^,^28^,^29^,^30^,^31^. Essentially, physic-chemical changes in the hydrogel polymer network and the movement of water and ions into and out of the polymer matrix occur due to stimuli. Stimuli-responsive hydrogel microlenses, which have a significant advantage over conventional microfluidic microlenses owing to their ability to undergo abrupt volumetric changes in response to their surrounding aqueous environment without the requirement of external controls and even power sources, were integrated into microfluidics, thus providing microfluidic systems with autonomous functionality^32^,^33^. However, the realization of an integrated system from optofluidic manipulation and a microfluidic channel is often hampered by the lack of a universal substrate for achieving monolithic integration. Specifically, out-of-plane optofluidic devices, with their optical axes perpendicular to the substrate, are compatible with conventional fixed micro-optical components and are one of the most mainly used optofluidic detection and analysis methods for projecting and imaging. The fabrication process of the precise alignment of optofluidic and microfluidic channels along the whole channel length in the perpendicular direction should be carried out with special attention and is usually of considerable complexity, thus restricting their use and diminishing potential applications. Therefore, an ingenious route to the rational design, fabrication and integration of both optofluidic and microfluidic channels is highly desired.

Here, we report on a UV-lithography technique for the integration of twin optofluidic and microfluidic channels together at relative positions on both sides of one substrate, which could be used to detect microfluidic channel through the reading of optofluidic signal. As an example, by using the twin optofluidic and microfluidic channels chip, we fabricate a hydrogel microlens in the optofluidic channel by using a femtosecond laser, which could adjust the focal length by using salt solutions with different concentrations. Imaging detection confirms that the images of microstructures at different depths in the adjacent microfluidic channel can be obtained via the optofluidic channel. The unique integration structure of optofluidic and microfluidic channels should provide a series of special functionalities to facilitate the development of diverse applications of the optofluidic chip.

## Results

**Figure 1:**
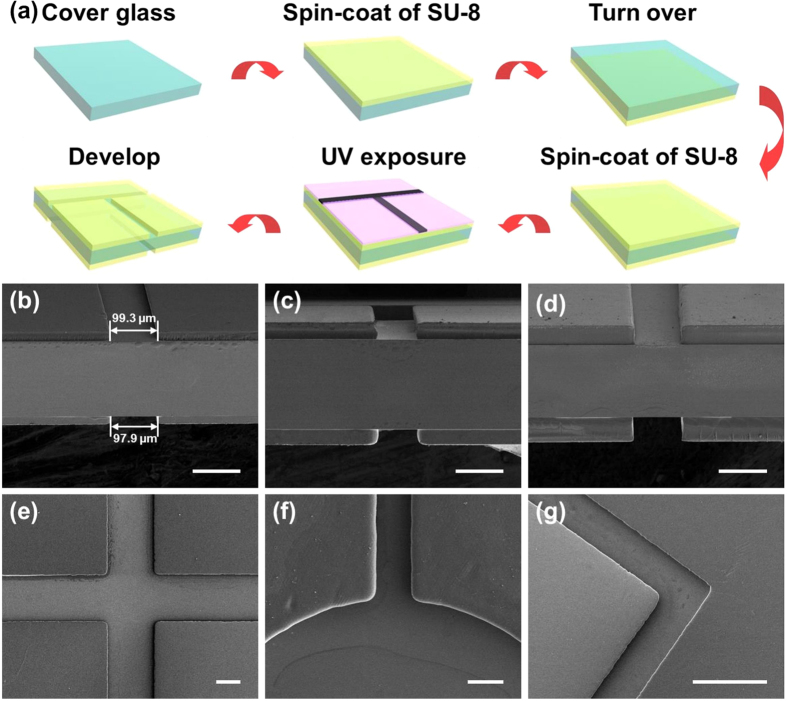
(**a**) Process flow of the twin optofluidic and microfluidic channels fabrication. Cross-section SEM images of twin microchannels with depths of 15 μm (**b**), 33 μm (**c**) and 45 μm (**d**), respectively. (**e–g**) SEM images of microchannels with different widths of 200 μm (**e**), 100 μm (**f**) and 50 μm (**g**), as well as various shapes: cross-shaped (**e**), spherical (**f**), orthogonal (**g**). Scale bar: 100 μm.

A one-time exposure method was used here to fabricate the twin optofluidic and microfluidic channels (Fig. 1a). Traditional two-side exposure method has been widely used to fabricate microfluidic chip and has advantage of the production of accurate non-symmetrical channel morphology. In this research, for the purpose of achieving the real detection, analysis and manipulation of the microobjects by using optofluidic system, an integrated chip with the symmetrical channel morphology up and down transparent substrate is necessary. The one-time exposure method can satisfy this requirement of chip, a transparent substrate (e.g., cover glass) is needed for light pass during lithography and let the optical signal pass through during the detection. So compared with the traditional two-side exposure method^34^, we provided the method which was more simple, flexible and convenient to fabricate aligned microchannels by UV exposure once, and during the fabrication, the method does not require the alignment process. SEM images of the twin microchannels are shown in Fig. 1b–g. The morphology of the microchannels was good, the side walls were perpendicular to the glass substrate and the rectangular cross-section boundary of the microchannels was clear. Microfluidic chips with different depths could be fabricated by adjusting the dilution ratio of SU-8 2050 with cyclopentanone and the rotation speed. The thicknesses of the SU-8 films were 15 μm, 33 μm and 45 μm, while the ratios of SU-8 2050 with cyclopentanone were 2:1, 5:1 and 10:1 by mass at the rates of 650 rpm, 650 rpm and 1000 rpm, respectively. Twin microchannels chips with the corresponding depths can be easily fabricated. SEM images of the cross-section of the fabricated chips are shown in Fig. 1b–d. In Fig. 1b, using a microchannel mask with a width of 100 μm, the measured widths of the upper and lower microchannels were 99.3 μm and 97.9 μm, respectively, showing a slight width mismatch of 1.4 μm between both microchannels. The slight width decrease of the lower microchannel may have been caused by light scattering loss through the glass during exposure. The mismatch in width of the twin microchannels of Fig. 1c,d were also less than 3 μm, accounting for only approximately 3% of the microchannel width, which did not affect the twin properties and the detection function brought by the twin microchannels. Because the preparation of the microchannels was a UV lithography process, various shapes of microchannels with different widths could be prepared by designing the corresponding mask patterns. Figure 1e–g show the SEM images of microchannels with widths of 200 μm, 100 μm and 50 μm, respectively. We injected different kinds of solutions into the twin microchannels respectively, and saw that the aligned twin microchannels on both surfaces of the coverslip can be controlled independently, meaning that different chemical or biological reactions could occur simultaneously in both microchannels without interfering with each other (Fig. S1 in supporting information).

In order to obtain a better morphology of twin microchannels chip, we investigated the influence of the exposure time on the fabrication. We fabricated twin microchannels chip with exposure time of 8 min, 10 min and 15 min, and characterized the morphology of the chips (Fig. S2 in supporting information), found that the polymerization degree of the SU-8 film on both sides of the coverslip was a little different. When the exposure time was 8 min, the SU-8 film on both sides of the coverslip could not be cross-linked well simultaneously, we can see some traces on the chip from the optical microscope image (Fig. S2a). And from the SEM images, the SU-8 film on the side near to the light source was cross-linked well, but it could be observed that the film on the far side was not polymerized sufficiently from the magnified SEM image (Fig. S2e). And if the exposure time was 15 min, from the optical microscope image, we can see that there was SU-8 photoresist residues in the microchannels (Fig. S2k). The SEM images showed that on the near side, SU-8 photoresist in the microchannels have also cross-linked and could not be developed in the SU-8 developer so the microchannels would have been blocked (Fig. S2l,m), and the film on the far side was cross-linked well (Fig. S2n,o). When the exposure time was 10 min, the twin microchannels were complete, and there were nearly no SU-8 photoresist residues in the microchannels. This was because that the coverslip used in the experiment was about 180 μm thick, much thicker than the SU-8 film, and the UV light would attenuate through the coverslip. During the one-time exposure process, SU-8 film on both sides of the coverslip should be cross-linked. So the exposure time used to fabricate the twin microchannels chip here was much longer than the twice of the exposure time to cross-link the film which has the same thickness. Zou *et al*. used a novel hybrid patterning technique based on hot embossing and inverse UV photolithography to fabricate microchannels and nanochannels simultaneously. In this work, 30 μm thick layer of SU-8 (MicroChem, SU-8 2015) was spin-coated onto the glass substrate with a thickness of 1 mm. After prebaked, the SU-8 layer was exposed to UV light from the back side of the glass substrate at the optical power density of 227.6 μW·cm^−2^ for 10 min to cross-link the photoresist^35^. So the exposure time has great influence on the morphology of the twin microchannels.

**Figure 2:**
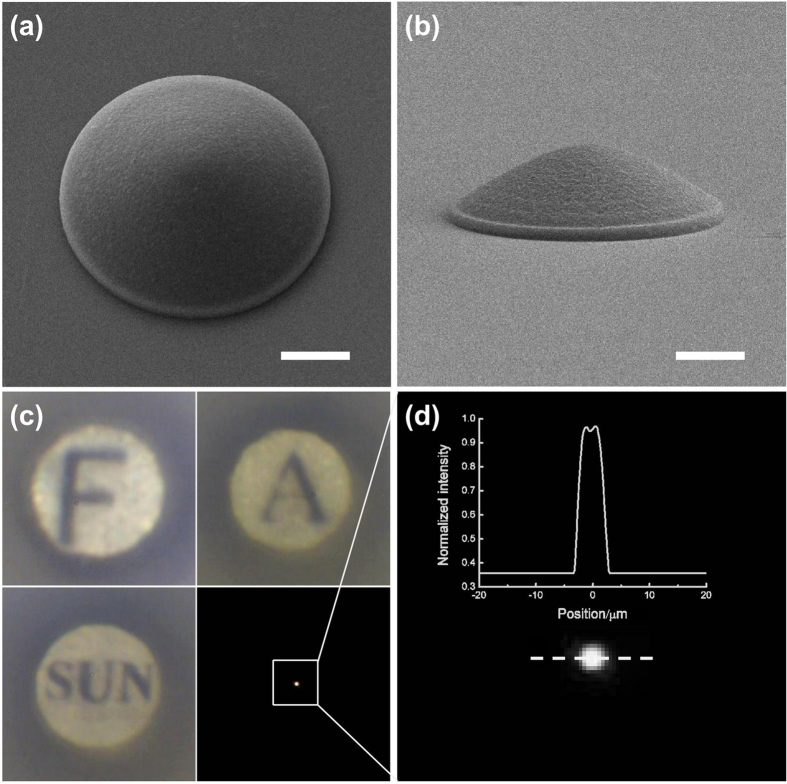
(**a**) SEM image of hyperbolic microlens from PEG-DA hydrogel with a radius of 10 μm and height of 5 μm. (**b**) Side-view image of the hyperbolic microlens. (**c**) Focusing test and imaging test of PEG-DA microlens in air. (**d**) Normalized light intensity distribution along the dotted line. Scale bar: 5 μm.

For optofluidic tunability of the optofluidic channel in the twin microchannels chip, a “smart” hydrogel, which can change shape and volume under various environment stimuli, was selected to fabricate the micro-optical component. Poly (ethylene glycol)s-based hydrogels have found widespread applications as biomaterials because of their particularly biocompatibility and has been studied as a component to fabricated tunable hydrogel microlens by some methods such as imprint lithography^36^. We choose the poly (ethylene glycol) diacrylate (PEG-DA) hydrogel here because of its property that it could swell when immersed in a water-ethanol solution of chloride salt. The PEG-DA monomer could be easily photocrosslinked when methylene blue (MB) is employed as a photosensitizer. A pinpointed femtosecond laser direct writing (FsLDW) was used to fabricate the PEG-DA hydrogel microstructures. This technology has shown a special capability for prototyping complicated 3D microstructures with nanometric resolution by using a wide range of photosensitive materials^37^,^38^,^39^,^40^. The laser power density used for fabrication was carefully optimized to improve the morphology of the PEG-DA hydrogel microstructures and we found that the surface smoothness was improved as the average laser power density reduced. The surface morphology of the hydrogel microstructures was better when the average laser power density was 4.5 mW∙μm^−2^ and the scanning step was 100 nm (Fig. S3 in supporting information). A PEG-DA hydrogel micro-optical component, i.e., a hyperbolic microlens with a radius of 10 μm and height of 5 μm, was fabricated by FsLDW (Fig. 2a), and the side view image was shown in Fig. 2b. The imaging capability and focusing capability were investigated and characterized, the optical imaging of letters “F”, “A” and “SUN” were shown as Fig. 2c. Under illumination by a halogen lamp, a bright focal spot was observed, Fig. 2d displayed the normalized light intensity distribution along a dotted line across the center of the focusing point, which indicates that the hydrogel microlens has very good optical property.

**Figure 3:**
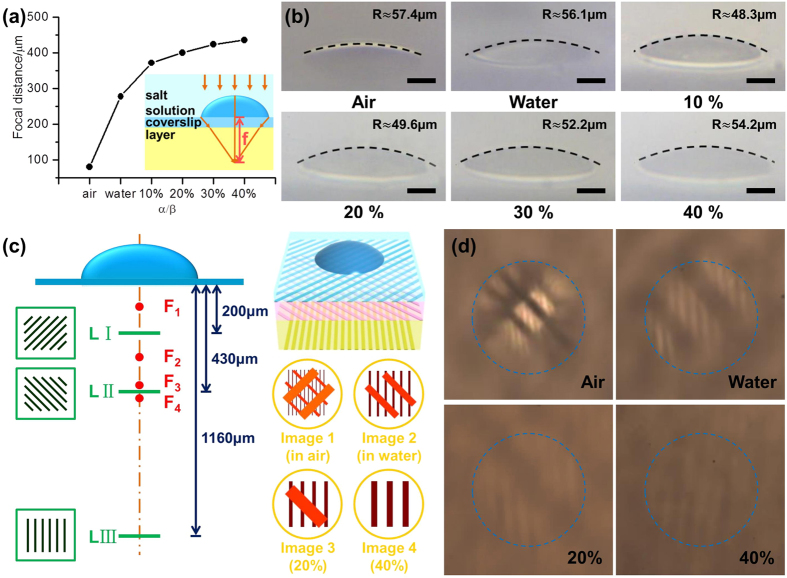
(**a**) Dependent curve of focal length on the stimuli solvents of different ratios of a CaCl_2_ aqueous solution, where α is the volume of the saturated CaCl_2_ aqueous solution and β is the total volume of the solution. (**b**) Side-view images of a hydrogel microlens with a radius of 20 μm and height of 8 μm after changing the surrounding solutions. Scale bar: 10 μm. (**c**) Schematic of the tunable imaging test of the PEG-DA microlens. LI, LII and LIII stand for three layers with the grating pattern below the microlens. F1, F2, F3 and F4 represent the focal point in air, water, 20% and 40% CaCl_2_ solution (left). Images 1–4 (bottom right) show the images formed by the microlens when changing the focal length. The corresponding optical microscopic pictures are shown in (**d**).

Taking advantage of the stimulus-response properties of the hydrogel materials, hydrogel-based microlenses present excellent optical tunability under external environment stimuli. When the PEG-DA microlenses were immersed into a water-ethanol solution of chloride salt, the solvent diffused into the hydrogel network could induce the microlenses to swell and the focal length to change. The tuning process of the hydrogel microlens was short and the focal length changed completely within a few minutes (Fig. S4). After the microlens immersed into solution for 5 minutes, the microlens swelled sufficiently, we measured the focal length of the PEG-DA hydrogel microlens in different ratios of CaCl_2_ aqueous solution, respectively. From the curve of Fig. 3a, it could be observed that the focal length of the PEG-DA microlens increased from 278 μm to 436 μm when the ratio of CaCl_2_ aqueous solution increased from 0 to 40%, and the focal length of the same microlens in air was 80 μm. When the hydrogel microlens was immersed into a water or CaCl_2_ water-ethanol solution, the microlens could swell due to the solution diffusing into the hydrogel network. By increasing the ratio of CaCl_2_ aqueous solution in the water-ethanol mixture, a larger swelled volume of the immersed microlens could be achieved due to the stronger infiltration from the surrounding mixture solution. Figure 3b shows the side view images of a PEG-DA hydrogel microlens under different ratios of CaCl_2_ aqueous solution in the water-ethanol mixture; an increasingly swelled volume could be observed as the ratio of the salt solution increased. Curvature radii of the swelled microlens in the surrounding mixture solution were calculated. The curvature radii of the hydrogel microlens in air and in water were approximately 57.4 μm and 56.1 μm, respectively. With increased ratio of the CaCl_2_ aqueous solution, the curvature radius increased from 48.3 μm at a 10% ratio to 54.3 μm at 40%. This was because the microlens expansion in the horizontal direction was restricted by the glass substrate, microlens expansion primarily along the lateral direction at first induced a decrease of the curvature radius. With increasing degree of hydrogel microlens expansion in the CaCl_2_ water-ethanol solution, the lateral expansion almost reached the maximum and expansion in the horizontal direction led to the curvature radius increasing slightly. We analyzed that the microlens swelled via solution diffusion, the refractive index of the microlens changed, which was the major reason for the focal length changing. When the ratio of CaCl_2_ aqueous solution became larger, we measured that the refractive index of the stimuli solvents also became larger. And after the microlens absorbed water and swelled, the refractive index of the hydrogel microlens will be smaller. So the effective refractive index difference between PEG-DA microlens and the outside environment solutions would be smaller as the ratio of CaCl_2_ aqueous solution increased. We calculated the effective refractive index difference between PEG-DA microlens and the outside environment solutions, the results were consist with the analysis (Table S1 in supporting information). Additionally, it is worth noting that the response was reversible and the focal length could be cyclically tuned by changing the surrounding solutions. We fabricated hydrogel microcubes, immersed and sonicated in water, and found that the adhesion between the substrate and the hydrogel microstructures was well (Fig. S5 and S6), so we think that the hydrogel microlens would not be destroyed and would not separate from the substrate when cyclically tuning the focal length.

To evaluate the tunability of the fabricated microlens, three transparent thin layers, of which one side is a grating pattern with a period of 50 μm, were placed below the hydrogel microlens at different distances. The angled line direction of the three grating patterns was not parallel to clearly observe the projection through the microlens. According to the focal lengths of the hydrogel microlens in different ratios of CaCl_2_ aqueous solution measured previously, the distances between the grating pattern and the microlens were 200 μm, 430 μm and 1160 μm, respectively. Figure 3c shows the schematic illustration of the test procedure. At first, the microlens was placed in air; the focal length was measured as 80 μm, and the images of all three grating patterns could be observed at the image plane because they were all placed outside the focal point. When the microlens was immersed into water, the focal length was increased to 278 μm, and only the images of two grating patterns were observed at the image plane; the grating pattern at 200 μm, which was nearest to the microlens, was inside the focal point. After stimulation of the chloride salt solution, the focal length of the PEG-DA microlens increased from 372 μm to 424 μm as the ratio of the CaCl_2_ aqueous solution increased from 10% to 30%; the grating pattern at 430 μm was always outside the focal point and similar results could be observed as in water. However, when approaching the focal point, the images of the grating patterns became larger and blurrier as the ratio of the CaCl_2_ aqueous solution increased. When the solution around the PEG-DA microlens was changed to 40% CaCl_2_ saturated aqueous solution and 60% ethanol, the focal length became 436 μm; only the image of the grating pattern at 1160 μm was in the image plane. All of the images are shown in Fig. 3d.

**Figure 4:**
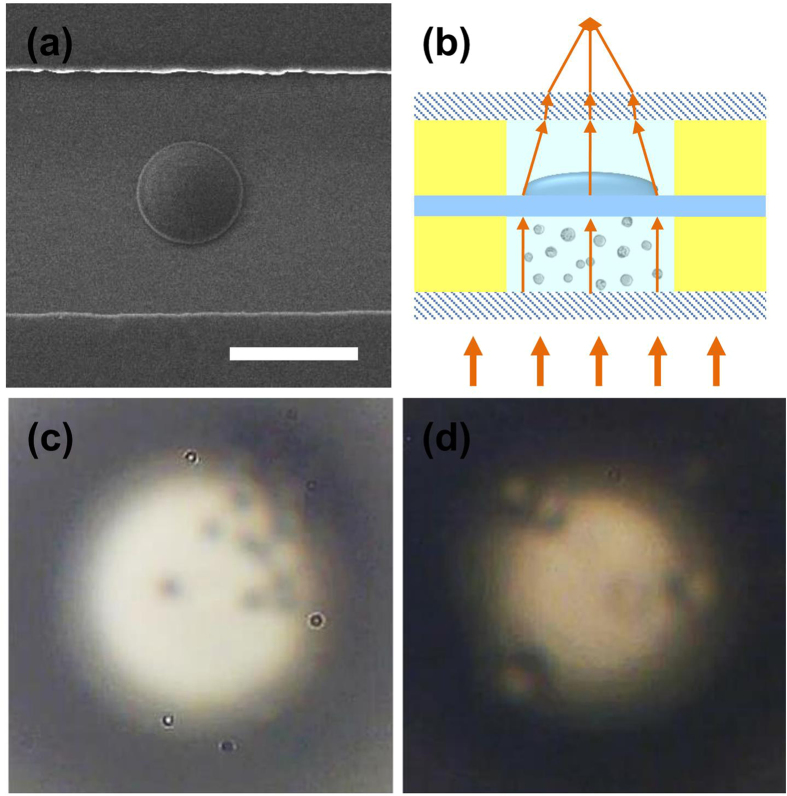
(**a**) SEM image of a hyperbolic microlens integrated within the optofluidic channel. Scale bar: 50 μm. (**b**) Schematic of the focusing characteristic resulting from the PEG-DA microlens in the integrated twin optofluidic and microfluidic channels. (**c**,**d**) Images of polystyrene spheres and HUVEC formed by a microlens using the twin optofluidic and microfluidic channels.

The tunable PEG-DA microlens could be easily integrated *in situ* into the optofluidic channel of the twin microchannels chip via FsLDW. A SEM image of a PEG-DA microlens inside a microchannel is shown in Fig. 4a. To further verify the imaging performance of the microlens and the utility of the twin microchannels, we used the PEG-DA microlens to observe polystyrene spheres with a radius of 5 μm and human umbilical vein endothelial cells (HUVEC) with a radius of approximately 15–20 μm in the microfluidic channel. Figure 4b is the schematic image of the cell or particle imaging using the PEG-DA microlens in an integrated twin microchannels chip. Using the twin microchannels chip, a transparent glass substrate was between the optofluidic channel and microfluidic channel, so it could not be avoided that optical interference and thermal interference could occur between the twin microchannels. The optical interference was expected in the experiment, because of the optical interference that we achieved the optical detection and optical imaging of microfluidic channel. The thermal interference caused by the optical interference was not expected in the experiment. But in the similar works, researchers used micro-optics to observe and focus on microobjects or live cells. Under illumination, the temperature of the surrounding environment would change because of the microlens focusing^41^,^42^. So we think that the temperature changed very little, and during the imaging process, the thermal interference has only a little influence on the result of the experiment. We injected the polystyrene spheres suspension and cell suspension into the microfluidic channel with a depth of approximately 30 μm. Figure 4c,d show the images of the polystyrene spheres and cells formed by the microlens in air, respectively. Because the focal length of the microlens (80 μm) was smaller than the distance between the microlens and upper surface of the microchannel (170 μm), as well as the thickness of the coverslip, images of all microparticles or cells below the microlens through the microchannel in the perpendicular direction could be collected. When the supporting environment was changed to water or a CaCl_2_ water-ethanol solution, the focal length of the microlens became larger than 278 μm, and the distance between the microlens and the lower surface of the microchannel was 200 μm; thus, no projection image was observed. If the microchannel was deep enough, we could adjust the focal length of the hydrogel microlens to realize the particle or cell imaging at different depths in the microfluidic channel.

The method of one-time exposure photolithography combined with FsLDW does not require a complex and precise alignment procedure. In addition to the integration of the microlens in the twin microchannels chip, the twin microchannels were highly promising for the integration of various passive and active micro-optical devices, including polymeric optical switches, wavelength division multiplexing, on-chip microlasers and optical amplifiers for lab-on-a-chip optofluidic applications. The integrated chip with twin optofluidic-microfluidic channels possesses many functions, e.g., detection, analysis, and physical manipulation, which greatly enhance the Lab-on-a-Chip functionalities for practical application.

## Discussions

The present work demonstrated that the twin optofluidic and microfluidic channels integrated in one chip have been fabricated successfully in the vertical direction of the substrate with exact alignment via one-time exposure photolithography. Flexibility and simplicity make the one-time exposure photolithography a powerful tool for twin microchannels integrated in one chip, and the twin microchannels with various scales and shapes have been easily fabricated. As a proof of concept, a hydrogel microlens was fabricated in the optofluidic channel, and the focal length of the hydrogel microlens by FsLDW was adjusted by adjusting the salt ion concentration of the environment solution. Images of microstructures at different depths were obtained by tuning the focal length of the microlens. By introduction various tunable micro-optical components within the optofluidic channel, the integrated chip with twin optofluidic-microfluidic channels possesses many functions, e.g., detection, analysis, and physical manipulation. We believe that this method will be applied to broader applications in the functionalization and miniaturization of Lab-on-a-Chip systems.

## Methods

### Twin optofluidic and microfluidic channels fabrication

The twin optofluidic and microfluidic channels were prepared using UV-lithography^35^. During the lithography process, aligned microchannels on both surfaces of the coverslip were fabricated simultaneously by performing only one UV exposure. The experimental details are shown as follows (Fig. 1a). Firstly, a coverslip was sonicated in acetone, absolute ethanol and deionized water for 30 min, respectively. Then the coverslip was dried in an oven at 95 °C for 5 h to completely evaporate water molecules from the substrates. Negative epoxy resin SU-8 2050 (Nano-Micro-Chem Company, American) diluted with cyclopentanone at the ratios of 2:1, 5:1 and 10:1 by mass and stirred with a magnetic stirrer for 24 h. The diluted photoresist was spin-coated on one surface of the coverslip for 60 s at different rotation speed. Subsequently, the photoresist was prebaked on an electronic hot plate to completely evaporate the solvent at 95 °C for 15 min and cooled to room temperature, it is worth noting that the time of prebaking would be properly and sufficiently. Then the coverslip was turned over and the previous process was repeated. In this process, no defects or film morphology deformation would occur, the films and the substrate had very good adhesion, this process didn’t lead to the detachment of the SU-8 film and the coverslip (Fig. S7). After finishing that, the microchannels on both surfaces of the coverslip were fabricated with an optical power density of 15 mW∙cm^−2^ and exposure time of 10 min. Because the SU-8 2050 photoresist is sensitive to ultraviolet light, ranging from 350 nm to 400 nm, here 365 nm was selected as the radiation wavelength. The twin microchannels chips with different widths and shapes could be fabricated by using different masks. After exposure, the sample was baked at 95 °C for 15 min, cooled to room temperature and developed in a SU-8 developer for 12 min to remove the unexposed areas on both surfaces, and rinsed in isopropyl alcohol for 15 s to remove the SU-8 developer. Finally, the aligned SU-8 microchannels were obtained, a cured PDMS slice was used to cover the SU-8 channel and pressed for adhesion.

### Hydrogel polymerization

Hydrogel microlenses in the optofluidic channel were fabricated using a home-made FsLDW system. The hydrogel prepolymer solution consisted of 100 μl of PEG-DA and 30 μl of MB (3 mg/ml) aqueous solution^43^,^44^. The mixture was sonicated in the dark for 5 min in a water bath to assure dissolution of all the chemicals and was used immediately. About 20 μl prepolymer solution was dropped onto the obtained twin microchannels and a small chamber of PDMS was used during the fabrication to minimize the effect of solution evaporation. The laser beam from a femtosecond laser (Spectra Physics 3960-X1BB, 80 MHz repetition rate, 120 fs pulse width and 780 nm central wavelength) was tightly focused by a 100× oil immersion objective lens with a high-numerical-aperture (Olympus, NA = 1.40) to directly write the hydrogel microlenses. After fabrication, the sample was rinsed in water several times to remove the unpolymerized prepolymer solution, according to the volumes of the microlenses, the prpolymer solution used for the fabrication was only a little part of the solution drop. Then the microlenses were obtained inside the optofluidic channel.

### Response measurement and optofluidic tunability of microlenses

According to the responsivity of the PEG-DA hydrogel, a water-ethanol solution of chloride salt was used to swell the hydrogel microlenses^45^. The solution consisted of a saturated CaCl_2_ aqueous solution and ethanol solution of CTAB (2 mg/ml), in which ethanol was used to accelerate the swelling process and enhance the diffusion of the saturated aqueous salt solution into the PEG-DA hydrogel, and a small amount of CTAB was used to improve the uniformity of the solution on the PEG-DA microlens surface. Different ratios of CaCl_2_ aqueous solution and ethanol were used as stimuli solvents for the focal length tuning. 10% of the CaCl_2_ aqueous solution was defined as a mixture of 10% (volume ratio) CaCl_2_ saturated aqueous solution and 90% ethanol.

### Characterization

The surface morphologies of the twin optofluidic and microfluidic channels and the hydrogel microstructures were characterized by using a field emission scanning electron microscope (SEM, JSM-7500F, JEOL, Japan). A thin layer of Au was sputtered onto the sample for better SEM imaging. Optical micrographs were taken by a Motic BA400 microscope and a charge-coupled device.

## Additional Information

**How to cite this article**: Lv, C. *et al.* Integrated optofluidic-microfluidic twin channels: toward diverse application of lab-on-a-chip systems. *Sci. Rep.* **6**, 19801; doi: 10.1038/srep19801 (2016).

## Supplementary Material

Supplementary Information
