# Supplementary material for: Integrated optofluidic-microfluidic twin channels: toward diverse application of lab-on-a-chip systems
Source: Sci Rep. 2016 Jan 29;6:19801. doi: 10.1038/srep19801 (PMC4731762; doi:10.1038/srep19801)
Supplement: Supplementary Information [file srep19801-s1.doc]

**Supporting Information**

**Integrated optofluidic-microfluidic twin channels: toward diverse application of lab-on-a-chip systems**

Chao Lv^1^, Hong Xia^1^, Wei Guan^1^, Yun-Lu Sun^1^, Zhen-Nan Tian^1^, Tong Jiang^1^, Ying-Shuai Wang^1^, Yong-Lai Zhang^1^, Qi-Dai Chen^1^, Katsuhiko Ariga^3,4^, Yu-De Yu^5^ and Hong-Bo Sun^1,2^

^1^State Key Laboratory on Integrated Optoelectronics, College of Electronic Science and Engineering, Jilin University, 2699 Qianjin Street, Changchun, 130012, People’s Republic of China.

^2^College of Physics, Jilin University, 119 Jiefang Road, Changchun, 130023, People’s Republic of China.

^3^International Center for Materials Nanoarchitectonics (MANA), National Institute for Materials Science (NIMS), 1-1 Namiki, Tsukuba, 305-0044 Japan

^4^Precursory Research for Embryonic Science and Technology (PRESTO) and Core Research for Evolutional Science and Technology (CREST), Japan Science and Technology Agency (JST), 4-1-8 Honcho, Kawaguchi, Japan

^5^State Key Laboratory on Integrated Optoelectronics, Institute of Semiconductors, Chinese Academy of Sciences, Beijing 100083, China

Correspondence and requests for materials should be addressed to Prof. Hong Xia (email: [hxia@jlu.edu.cn](mailto:hxia@jlu.edu.cn) ) or Prof. Hong-Bo Sun (email: [hbsun@jlu.edu.cn](mailto:hbsun@jlu.edu.cn) )

**Materials and equipment**

The poly (ethylene glycol) diacrylate (PEG-DA, average Mn 575), methylene blue (MB) and cetyltrimethyl-ammonium bromide (CTAB) were purchased from Sigma-Aldrich and used without further purification. The cyclopentanone (analytically pure) used to dilute SU-8 2050 and the calcium chloride anhydrous (CaCl_2_, analytically pure), ethanol (analytically pure) used to prepare the water-ethanol solution was purchased from Beijing chemical works. All the water used in the experiment was deionized. The SU-8 films with different thicknesses were obtained using a spin-coater (KW-4A, Institute of microelectronics of Chinese academy of sciences, China), prebaked and post exposure baked using an electronic hot plate (CT-946, CTBRAND, American). The twin microchannels chips were fabricated by using an UV lithography machine (JKG-2A, Shanghai optical instrument factory, China).

**Flow tests**

The aligned twin microchannels on both surfaces of the coverslip can be controlled independently. Optical microscope images of an empty twin microchannels chip with a width of 100 µm and depth of 40 µm are shown in Fig. S1a (top view). When a solution of Rhodamine B (RB) was injected into the upper microchannel via capillary force, a pink color microchannel image was represented (Fig. S1d). After a solution of MB was injected into the symmetrical lower microchannel of the empty twin microchannels chip, a blue color microchannel could be observed (Fig. S1g). When we simultaneously injected the two types of solutions into the two microchannels, i.e., the pink color solution in the upper microchannel and the blue color solution in the lower microchannel, the purple color of the twin microchannels was observed (Fig. S1j). To make the result more clear, images of the cross-section of the microfluidic chips and the fluorescence images are shown. From Fig. S1b, it can be easily observed that both the microchannels were initially empty. When we injected the RB solution into the upper microchannel, the lower microchannel and both of the twin microchannels, respectively, the locations of the corresponding microchannels were dark, representing that the microchannels were full of solution (as shown in Fig. S1e, S1h and S1k). For the fluorescence images, we can see a blue emission from the SU-8 side walls of the twin microchannels chip and bright red fluorescence from RB in the corresponding microchannel under a 405 nm laser illumination (Fig. S1f, S1i and S1l).


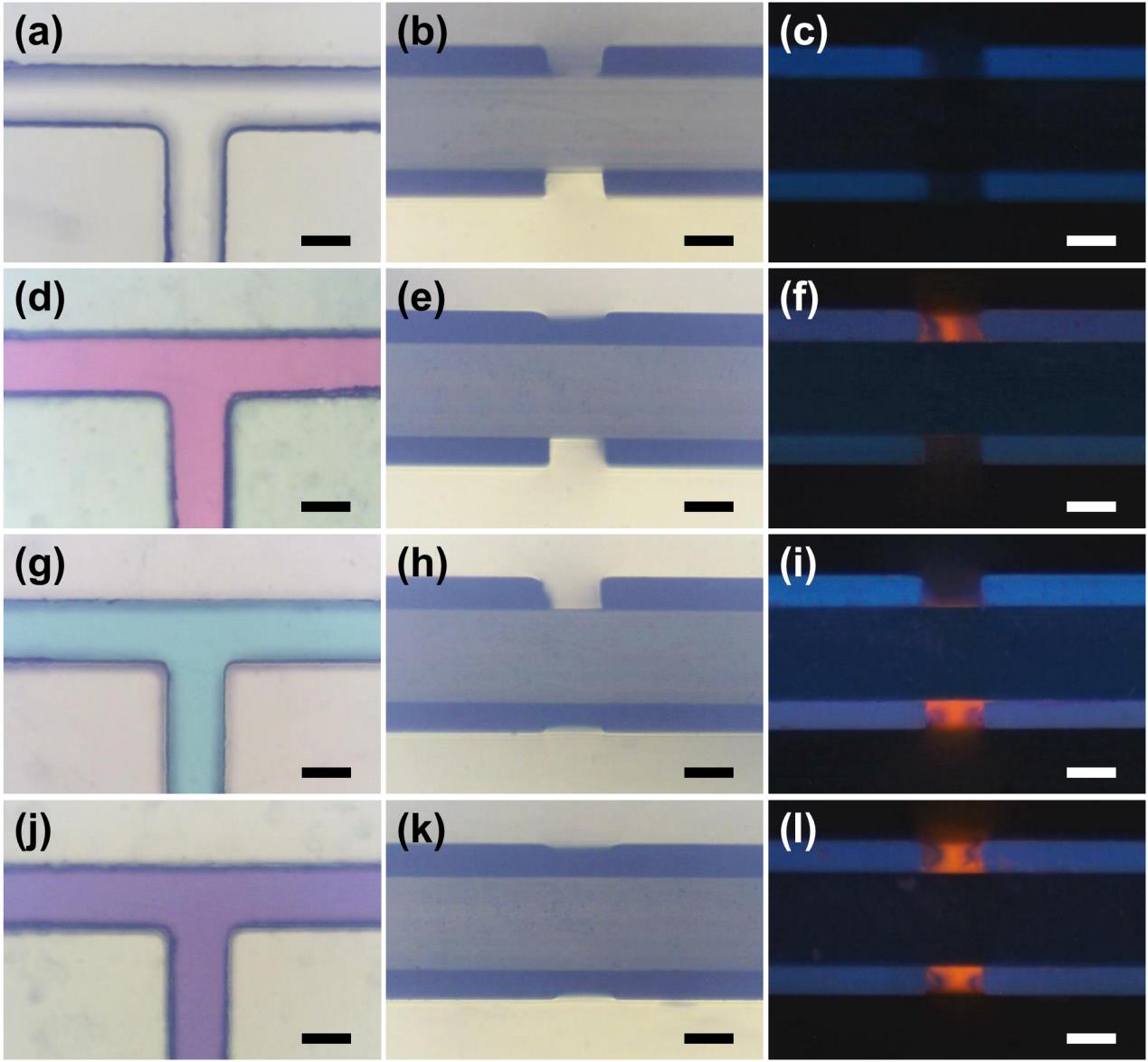


**Figure S1.** Optical microscopic images of the flow tests. Top view (a), cross-section (b) and fluorescent microscopy (c) of empty twin microchannels with a width of 100 µm and depth of 40 µm. Top view (d), cross-section (e) and fluorescent microscopy (f) of the twin microchannels with a RB solution injected into the upper microchannel. Top view (g) of the twin microchannels with a MB solution injected into the lower microchannel. Cross-section (h) and fluorescent microscopy (i) of the twin microchannels with the RB solution injected into the lower microchannel. Top view (j) of the twin microchannels with the RB and MB solution injected into the two microchannels, respectively. Cross-section (k) and fluorescent microscopy (l) of the twin microchannels with the RB solution injected into the two microchannels. Scale bar: 100 µm.


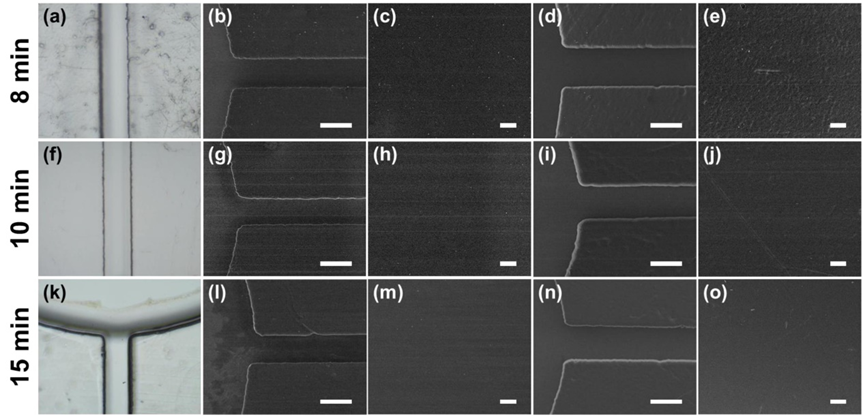
**Figure S2.** Optical microscope images and SEM images of the microchannels chips with different exposure time. (a) Optical microscopic images of twin microchannels chip with exposure time of 8 min. (b-e) SEM images of twin microchannels chip with exposure time of 8 min, b-c were images of the SU-8 film on the side near to the light source and d-e shown on the far side, c and e were the magnified detail of b and d, respectively. The SU-8 layer didn’t cross-link well. (f-j) Optical microscope images and SEM images of twin microchannels chip with exposure time of 10 min, h and j were the magnified detail. (k-o) Optical microscope images and SEM images of twin microchannels chip with exposure time of 15 min, SU-8 photoresist residues in the microchannels and the microchannels had been blocked. Scale bar: 100 µm.

**Optimization of FsLDW processing parameters**

To obtain excellent morphology of the hydrogel microstructure, the laser power density should be carefully optimized. For the fabricated cube (Fig. S3a), the surface smoothness was improved as the average laser power density reduced from 18.5 mW∙µm^-2^ to 4.5 mW∙µm^-2^ when the scanning step was 100 nm. Through a preprogrammed 3D design, the PEG-DA can be photopolymerized into various complicated 3D geometries by FsLDW, e.g., well-shaped microstructures of pentagram, hexagon, cylinder and ring forms were achieved when the average laser power density was 4.5 mW∙µm^-2^ (Fig. S3b). As shown in the pictures, the surfaces of the as-prepared microstructures were smooth enough for optical applications.


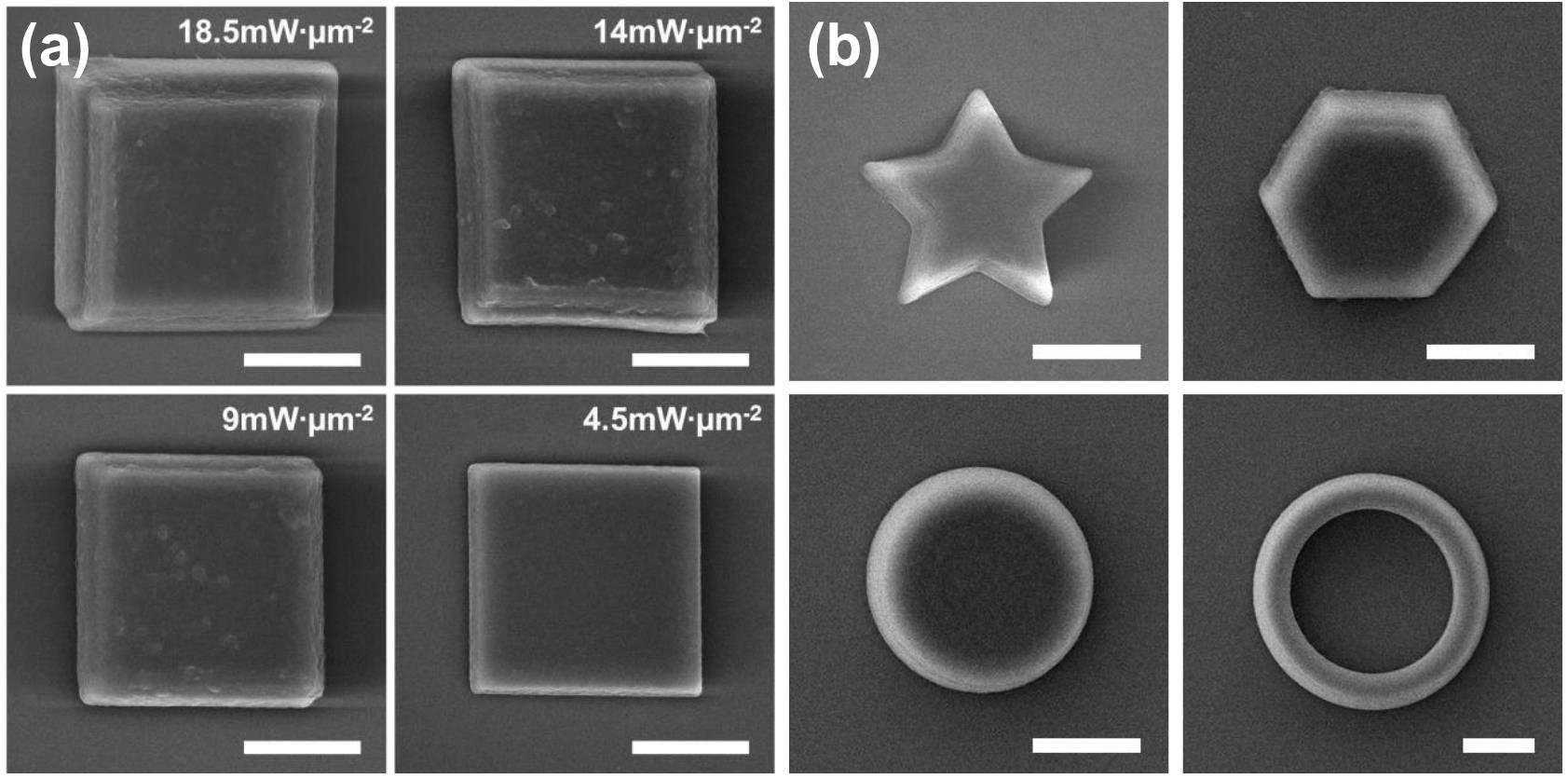


**Figure S3.** (a) SEM images of PEG-DA hydrogel microcubes fabricated with different average laser power densities with a scanning step of 100 nm. (b) SEM images of PEG-DA hydrogel microstructures with different geometries. Scale bar: 5 µm.


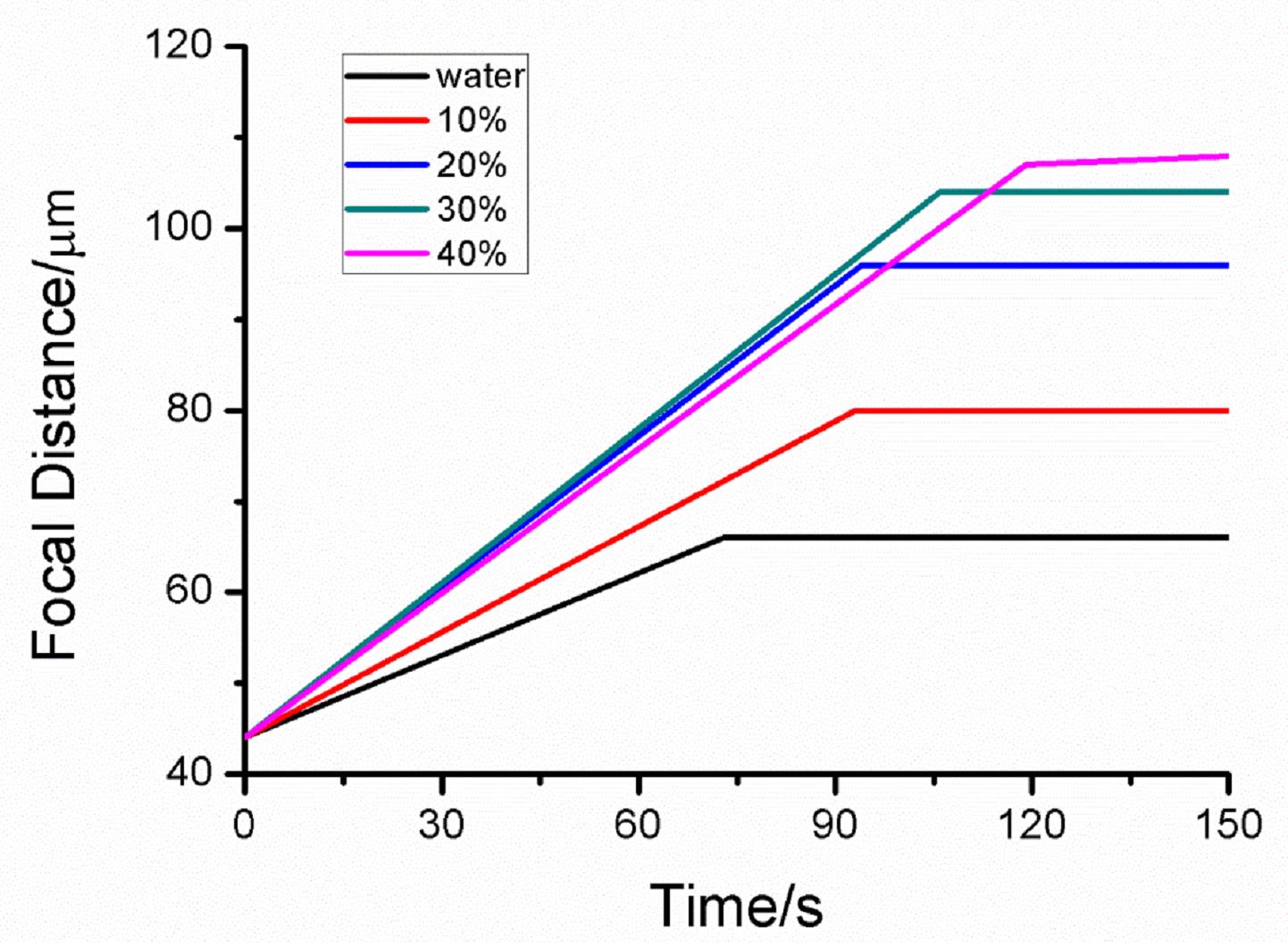


**Figure S4.** Dependent curve of focal length on time when hydrogel microlens immersed into water-ethanol solution of chloride salt from the air.

**The effective refractive index difference**

When the PEG-DA hydrogel microlens was immersed into a water-ethanol solution of chloride salt, the focus length *f* can be calculated by:

*f = n_solution_/[(n_hydrogel_ – n_solution_)/R +(n_glass_ - n_hydrogel_)/R’]*

which *R* and *R’* represent the two different curvature radii of the hydrogel microlens, and n_hydrogel_, n_solution_, and n_glass_ represent the refractive indices of PEG-DA hydrogel, water-ethanol solution of chloride salt and glass, respectively. As the hydrogel microlens was attracted on the substrate, *R’ =∞*, the formula can be simplified as ：

*f = n_solution_ R/(n_hydrogel_ – n_solution_)*

Therefore, the effective refractive index between PEG-DA microlens and outside environment could be calculated by:

*n_hydrogel_ – n_solution_ = n_solution_ R/ f*

Using the *R*, *f* and the refractive indices of different ratios of CaCl_2_ aqueous solution measured in the experiment, we calculatd the *n_hydrogel_ – n_solution_*, the results were shown in Table S1.

|  | *n_solution_* | *R**/μm* | *f/μm* | *n_hydrogel_-n_solution_* |
| --- | --- | --- | --- | --- |
| Water | 1.3311 | 56.1 | 278 | 0.268614 |
| 10% | 1.3743 | 48.3 | 372 | 0.178437 |
| 20% | 1.3863 | 49.6 | 400 | 0.171901 |
| 30% | 1.4065 | 52.2 | 424 | 0.171669 |

**Table S1.** The effective refractive index difference between PEG-DA microlens and the water-ethanol solution of chloride salt.

**
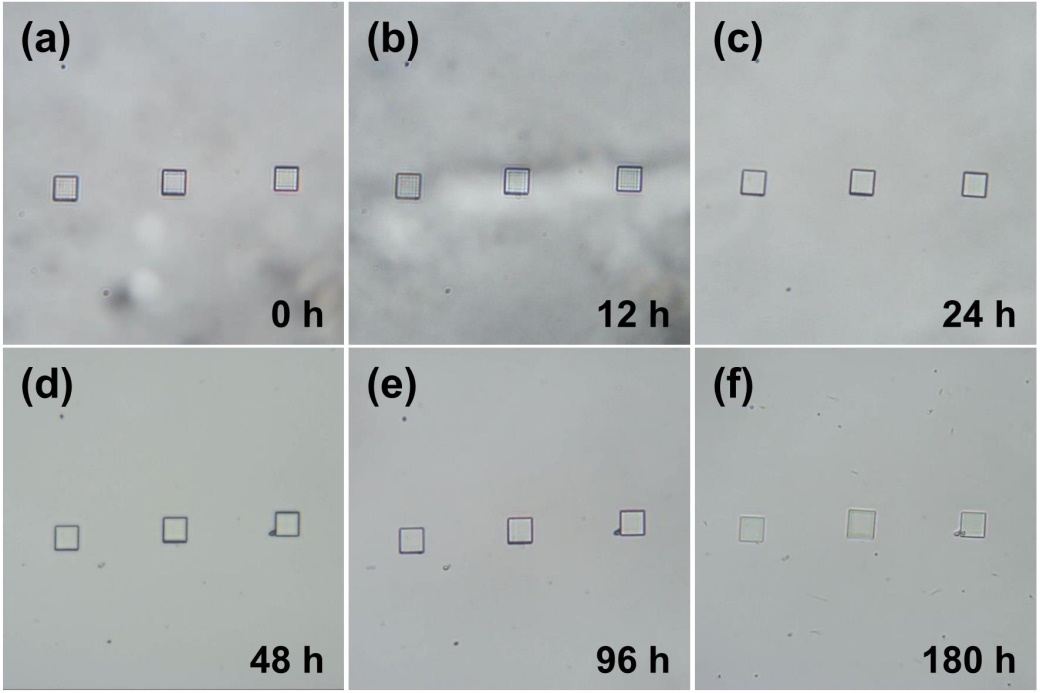
**

**Figure S5.** Optical microscopic images of microcubes immersed in water for different time. After immersion for 180 h, the microcubes were not destroyed and didn’t separate from the substrate.


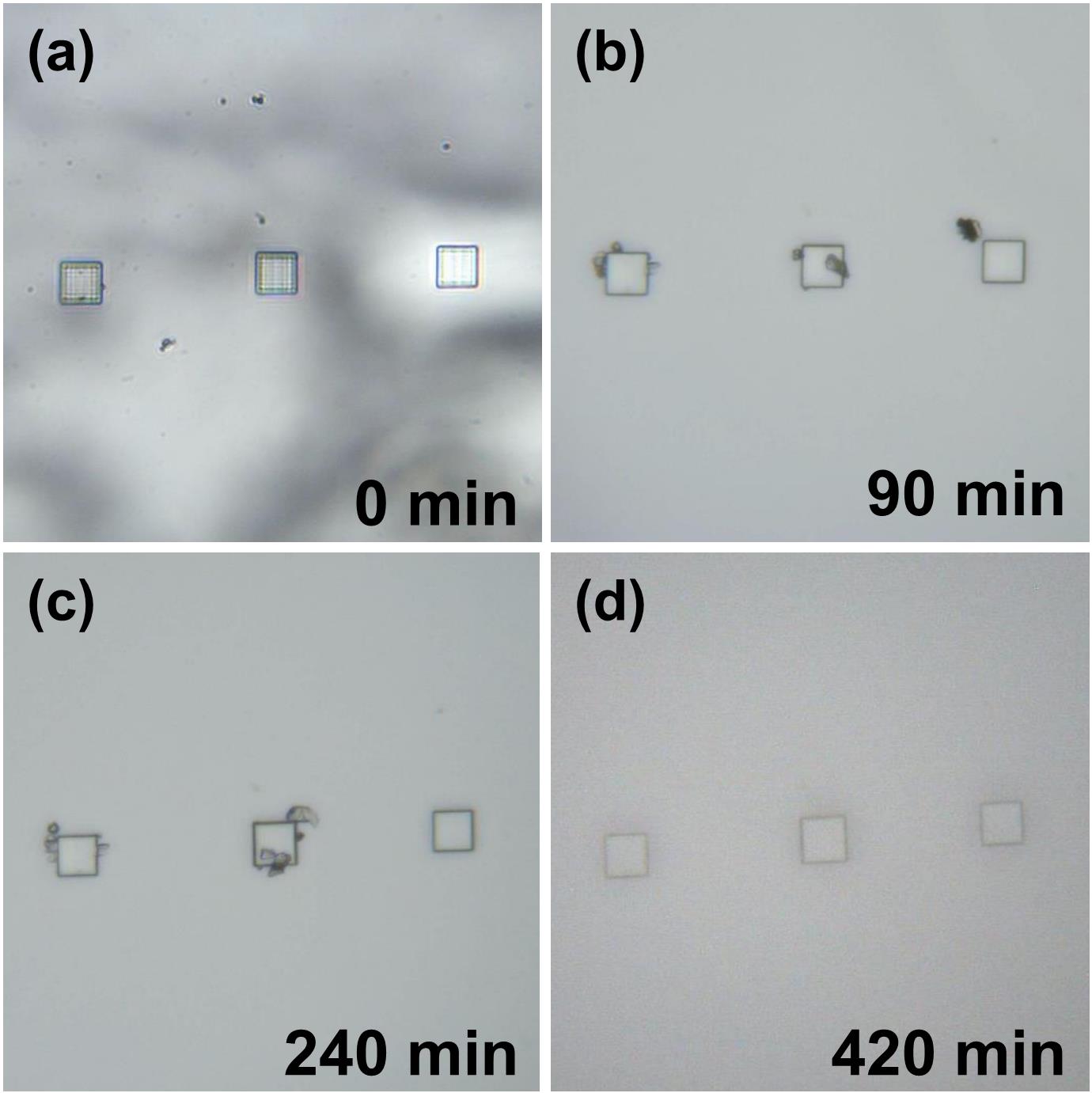


**Figure S6.** Optical microscopic images of microcubes sonicated in water for different time. After 420 min sonication in water with the frequency of 40 kHz and the power of 100 W, the microcubes were still complete.


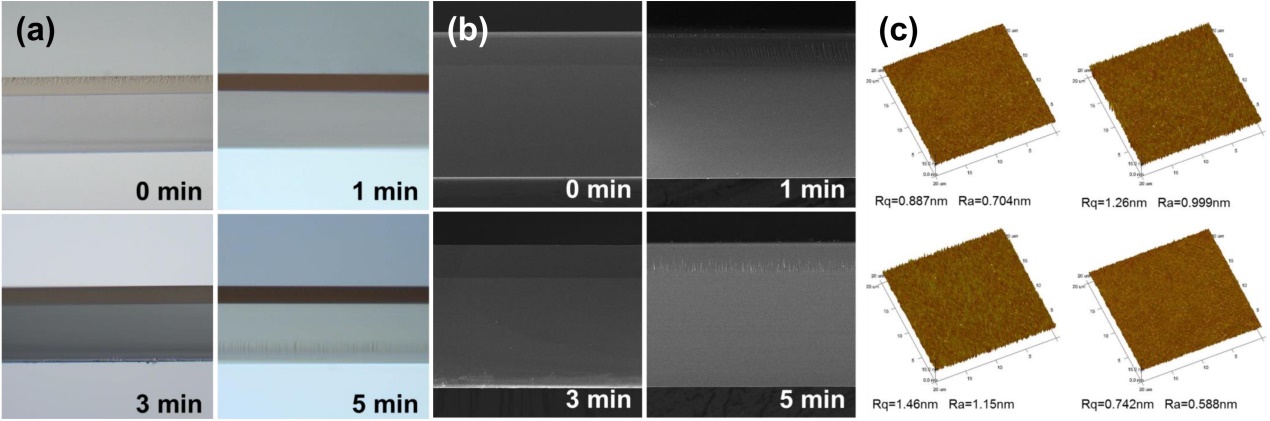


**Figure S7.** The film morphology after placed on the vacuum grip in the second spin-coating process. (a-b) Optical microscopic images and SEM images of the cross-section of the SU-8 film after prebaking (the first one) and the samples which have been placed on the vacuum grip for 1 min, 3 min and 5 min, respectively. (c) AFM images of the surfaces morphology of SU-8 film after vacuum gripped.
